# Immunophenotyping characteristics and clinical outcome of COVID-19 patients treated with azvudine during the Omicron surge

**DOI:** 10.3389/fimmu.2024.1465238

**Published:** 2024-11-25

**Authors:** Meihua Qiu, Xiaogang Song, Qianqian Zhang, Shenchun Zou, Lingling Pang, Xueyuan Nian

**Affiliations:** ^1^ Department of Respiratory and Critical Care Medicine, Qingdao University Medical College Affiliated Yantai Yuhuangding Hospital, Yantai, China; ^2^ Department of Respiratory and Critical Care Medicine, the Second Affiliated Hospital of Dalian Medical University, Dalian, China; ^3^ Department of Imaging, Qingdao University Medical College Affiliated Yantai Yuhuangding Hospital, Yantai, China; ^4^ Department of Gastroenterology, Qingdao University Medical College Affiliated Yantai Yuhuangding Hospital, Yantai, China

**Keywords:** lymphocyte subsets, CD4^+^ T cell, COVID-19, azvudine, mortality

## Abstract

**Background:**

Little is known about immunophenotyping characteristics and clinical outcomes of COVID-19 patients treated with azvudine during the Omicron variant surge.

**Methods:**

This study enrolled patients diagnosed with COVID-19 from December 2022 to February 2023. The primary outcome was defined as all-cause mortality, along with a composite outcome reflecting disease progression. The enrolled patients were followed for a period of 60 days from their admission.

**Results:**

A total of 268 COVID-19 patients treated with azvudine were enrolled in this retrospective study. The study found that the counts of lymphocyte subsets were significantly reduced in the composite outcome and all-cause mortality groups compared to the non-composite outcome and discharge groups (all *p* < 0.001). Correlation analysis revealed a negative association between lymphocyte subsets cell counts and inflammatory markers levels. The receiver operating characteristic (ROC) curve analysis identified low CD4^+^ T cell count as the most significant predictor of disease progression and all-cause mortality among the various lymphocyte subsets. Additionally, both the Kaplan-Meier curve and multivariate regression analysis demonstrated that low CD4^+^ T cell count level (< 156.00 cells/μl) was closely associated with all-cause mortality in COVID-19 patients treated with azvudine.

**Conclusions:**

A low CD4^+^ T cell count may serve as a significant predictive indicator for identifying COVID-19 patients receiving azvudine treatment who are at an elevated risk of experiencing adverse outcomes. These findings may offer valuable insights for physicians in optimizing the administration of azvudine.

## 1 Introduction

The emergence of severe acute respiratory syndrome coronavirus 2 (SARS-CoV-2) in late 2019 rapidly led to a global pandemic of respiratory illness, known as coronavirus disease 2019 (COVID-19) (1). As of October 22, 2023, the World Health Organization (WHO) reported a cumulative total of 770 million confirmed COVID-19 cases globally, resulting in approximately 6.97 million deaths. Over the past few years, the SARS-CoV-2 virus has undergone several mutations, resulting in the emergence of five major variants: alpha (B.1.1.7), beta (B.1.351), gamma (P.1), delta (B.1.617.2), and omicron (B.1.1.529) (2). The WHO designated Omicron as a variant of concern (VOC) on November 26, 2021, and it has subsequently emerged as the predominant strain globally (3). Compared to earlier variants, the Omicron variants were featured with higher transmissibility and more striking antibody evasion (4). Though the majority of individuals infected with Omicron tend to experience milder symptoms, result to lower rates of hospitalization and mortality, the vulnerable individuals—including the elderly and those with underlying comorbidities—tent to experience a more unfavorable prognosis than the general population (5, 6).

The innate and adaptive immune responses activated by SARS-CoV-2 infection are crucial for the clearance of invading virus. However, uncontrolled inflammatory innate immune responses and impaired adaptive immune responses can result in a cytokine storm and a state of hyperinflammation status, then lead to harmful tissue damage both locally and systemically (7). Accumulating data indicating that lymphopenia is a significant laboratory finding frequently observed in hospitalized COVID-19 patients (8–10), and it is recognized as a reliable prognostic marker for adverse outcomes related to disease severity (11–15). As the novel coronavirus continues to mutate, the prognostic significance of lymphocyte subsets remains uncertain, particularly in the context of the Omicron variant.

The Chinese government announced that COVID-19 patients did not need to be quarantined since December 2022. A significant proportion of people were infected with SARS-CoV-2 in the next several months during the Omicron surge. During this period, the demand for antivirus drugs among COVID-19 patients surpasses the current supply. Azvudine, a domestically developed oral antiviral agent, was the first approved RNA-dependent RNA polymerase (RdRp) inhibitor in China (16). On July 25, 2022, the National Medical Products Administration granted conditional authorization for the use of azvudine in treating COVID-19. Current clinical evidence suggests that the administration of azvudine has demonstrated a significant reduction in in-hospital mortality rates among patients with COVID-19, particularly within the severe and critical subgroups (17–19). However, there is a lack of reports on the lymphocyte subpopulations profile that may predict disease progression and mortality in COVID-19 patients receiving azvudine treatment. Therefore, the present study aims to explore the clinical manifestations and immunophenotyping characteristics of COVID-19 patients who received azvudine treatment during the period of Omicron variant prevalence. Additionally, our objective is to provide valuable insights to optimize the use of azvudine.

## 2 Materials and methods

### 2.1 Study design and participants

The retrospective study was conducted at the Qingdao University Medical College Affiliated Yantai Yuhuangding Hospital, China, from December 15, 2022, to February 28, 2023. The inclusion criteria for the study were as follows: a) all patients who tested positive for SARS-CoV-2 infection via Real Time-Polymerase Chain Reaction (RT-PCR), b) CT imaging findings that met the criteria for viral pneumonia, and c) all the patients received azvudine treatment and underwent peripheral blood lymphocyte subset testing via flow cytometry. The exclusion criteria included: a) individuals under the age of 18, b) patients who received additional antiviral drugs, such as Paxlovid, alongside azvudine, c) those for whom flow cytometry measurement was not performed, and d) patients with incomplete data. At present, according to the Diagnosis and Treatment Program for Novel Coronavirus Pneumonia (ninth Edition) (20), the hospitalized COVID-19 patients were categorized into three groups based on the severity of their condition: moderate, severe, and critical. The present study was approved by the ethics committee of Yantai Yuhuangding Hospital (no. 2024521), and the requirement for informed consent was waived due to the retrospective study design.

### 2.2 Data collection

The enrolled patients were administered azvudine treatment upon admission to the hospital and received additional treatments based on medical professionals’ discretion. These therapies included systemic corticosteroids, antibiotics, anticoagulants, immunoglobulin, supplemental oxygen including nasal catheter for oxygen/face mask oxygen inhalation, high-flow oxygen/noninvasive ventilation (HF/NIV), and invasive mechanical ventilation (IMV). Data derived from the electronic health records of COVID-19 included age, gender, clinical manifestations, medical history (e.g., comorbidities), imaging data, treatment regimens, clinical outcomes, and laboratory findings. The laboratory findings included lymphocyte subset parameters, interleukin-6 (IL-6), ferritin, C-reactive protein (CRP), procalcitonin (PCT), D-dimer, among others, and were conducted upon admission. Following the collection of blood specimens, azvudine administration was initiated. Lymphocyte subset analysis was conducted on patients utilizing antibodies and a cell analyzer supplied by Becton, Dickinson and Company.

### 2.3 Outcomes

The primary outcome was defined as all-cause mortality, along with a composite outcome of disease progression including symptom and CT finding aggravation, receiving more higher level of oxygen treatment, admission to the intensive care unit (ICU), and all-cause mortality. The secondary outcomes encompassed each of the individual disease progression. Patient outcomes were documented from admission until the occurrence of outcome events, discharge, or death, whichever occurred first. The discharge criteria included viral nucleic acid shedding and stable condition of any concurrent diseases.

### 2.4 Follow-up

All enrolled patients were followed for a period of 60 days from their admission, utilizing medical records and telephone consultations as data sources. This study primarily focused on the survival status throughout the 60-days follow-up period, with particular emphasis on accurately documenting the time of mortality.

### 2.5 Statistical analysis

Descriptive statistics were conducted to summarize all variables in the study. All continuous variables were presented as median (25th–75th percentile) and compared with the Mann–Whitney U test, while categorical variables were presented as numbers (%) by χ2 test or the Fisher’s exact test. Spearman rank correlation coefficient was employed to examine the associations between lymphocyte subsets and clinical indicators. A receiver operating characteristic (ROC) curve was plotted to determine the area under the curve (AUC), as well as the sensitivity and specificity of lymphocyte subset parameters. The association between the indicators and outcomes was estimated using Kaplan-Meier analysis and a multivariate logistic regression model. Statistical analyses were performed using SPSS 18.0 software (IBM Corp., Armonk, NY, USA) and GraphPad Prism 8.0 software (GraphPad Software Corp, San Diego, California, USA). A two-sided *p* < 0.05 was statistically significant.

## 3 Results

### 3.1 Demographic and clinical characteristics of enrolled COVID-19 patients

Following established inclusion and exclusion criteria, data were ultimately collected and analyzed information from a cohort of 268 COVID-19 patients. Flow cytometry measurements were performed on all patients, followed by administration of azvudine treatment, and the patients were followed over a period of 60 days. The demographic, clinical characteristics, treatments regimens and outcomes of the participants were presented in **Table 1**. The median age of the 268 enrolled patients was 72.00 (64.00–81.00) years, with a predominance of male patients (n = 174, 64.90%). Among the cohort, 50 patients reported a history of smoking history. The majority of participants had preexisting medical conditions, with hypertension (41.42%), diabetes mellitus (27.99%), coronary artery disease (19.78%), chronic pulmonary disease (13.43%), and cancer (7.46%) being the most prevalent. Chronic pulmonary diseases included asthma, chronic obstructive pulmonary disease, interstitial lung disease, and bronchiectasis. The proportion of enrolled patients classified as severe or critical was 42.91%. Notably, 207 (77.24%) received oxygen therapy. Systemic corticosteroids were administered to 247 patients (92.16%), while antibiotics were prescribed to 226 individuals (84.33%), and anticoagulants were utilized in 148 cases (55.22%). A minority of patients, specifically 17 individuals (6.34%), underwent immunoglobulin therapy.

**Table 1:** Demographics and clinical characteristics of COVID-19 patients, according to disease progression and clinical outcome.

	All (n = 268)	Composite outcome (n = 66)	Non-composite outcome (n = 202)	*p*-Value	Death (n = 37)	Discharge (n = 231)	*p*-Value
Age (years)	72.00(64.00–81.00)	76.00(65.50–85.00)	72.00(64.00–81.00)	0.011	82.00(70.00–86.00)	72.00(64.00–80.00)	0.002
Female, n (%)	94(35.07)	19(28.79)	75(37.13)	0.218	13(35.14)	81(35.06)	0.993
Smoking history, n (%)	50(18.66)	16(24.24)	34(16.83)	0.180	10(27.03)	40(17.32)	0.159
Hospital time (days)	8.00(6.00–11.00)	12.14(7.00–13.75)	8.00(6.00–10.00)	< 0.001	10.00(6.00–14.00)	8.00(6.00–10.00)	0.031
PaO2 (mmHg)	81.25(65.20–103.75)	73.20(62.60–99.30)	82.65(70.08–105.60)	0.069	73.90(63.90–96.70)	81.95(66.88–105.20)	0.167
PaO2/FiO2 (mmHg)	296.58(202.68–366.78)	204.24(171.20–254.00)	302.10(213.75–361.43)	< 0.001	208.50(170.7–254.00)	306.10(218.00–375.50)	0.002
Comorbidity, n (%)							
Hypertension	111(41.42)	17(25.76)	94(46.53)	0.003	17(45.95)	94(40.69)	0.547
Diabetes mellitus	75(27.99)	23(34.85)	52(25.74)	0.153	12(32.43)	63(27.27)	0.516
Coronary disease	53(19.78)	15(22.73)	38(18.81)	0.488	8(21.62)	45(19.48)	0.761
Chronic pulmonary disease	36(13.43)	19(28.79)	17(8.42)	< 0.001	11(29.73)	27(11.69)	0.003
Cancer	20(7.46)	9(13.64)	11(5.45)	0.028	5(13.51)	15(6.49)	0.131
Severity, n (%)							
Moderate	153(57.09)	19(28.79)	134(66.34)	< 0.001	5(13.51)	148(64.07)	< 0.001
Severe	80(29.85)	26(39.39)	54(26.73)	0.051	16(43.24)	64(27.71)	0.055
Critical	35(13.06)	21(31.82)	14(6.93)	< 0.001	16(43.24)	19(8.23)	< 0.001
Oxygen support, n (%)							
NO	61(22.76)	10(15.15)	51(25.25)	0.089	0(0)	61(26.41)	< 0.001
NC/FM	192(71.64)	57(86.36)	135(66.83)	0.002	33(89.19)	24(10.39)	< 0.001
HF/NIV	59(22.01)	43(65.15)	16(7.92)	< 0.001	37(100.00)	22(9.52)	< 0.001
TI	21(7.84)	21(31.82)	0(0)	< 0.001	16(43.24)	5(2.16)	< 0.001
Medication, n (%)							
Systemic steroid	247(92.16)	66(100.00)	181(89.60)	0.003	37(100.00)	210(90.91)	0.090
Antibiotics	226(84.33)	66(100.00)	160(79.21)	< 0.001	37(100.00)	189(81.82)	0.002
Anticoagulants	148(55.22)	49(74.24)	99(49.01)	< 0.001	31(83.78)	117(50.65)	< 0.001
Immunoglobulin	17(6.34)	17(25.76)	0(0)	< 0.001	15(40.54)	2(0.87)	< 0.001

PaO2/FiO2, arterial partial pressure of oxygen/fraction of inspired oxygen; NO, no oxygen inhalation; NC/FM, nasal catheter for oxygen/face mask oxygen inhalation; HF/NIV, high-flow oxygen/noninvasive ventilation; TI, tracheal intubation.

The COVID-19 patients were categorized into two distinct groups based on a composite outcome of disease progression: namely the composite outcome group (n = 66, 24.63%) and the non-composite outcome group (n = 202, 75.37%) (**Table 1**). Notable differences were observed between the two groups concerning age, duration of hospitalization, and arterial partial pressure of oxygen (PaO2)/fraction of inspired oxygen (FiO2) levels (all *p* < 0.05). Furthermore, the prevalence of hypertension, chronic pulmonary disease, and cancer among hospitalized patients was significantly higher in the composite outcome group compared to the non-composite outcome group (all *p* < 0.05). The occurrence of critical COVID-19 cases was also significantly higher in the composite outcome group compared to the non-composite outcome group (all *p* < 0.05). However, no statistically significant difference was found in the incidence of severe COVID-19 (*p* = 0.051). Although the *p*-value was not < 0.05, there was a trend that the severe type of COVID-19 was more common in composite outcome group. It is posited that an increase in the sample size could yield statistically significant results. Based on medical records, all patients received systemic steroid and antibiotics treatment in the composite outcome group. In addition, 49 (74.24%) patients received anticoagulants therapy and 17 (25.76%) patients received immunoglobulin therapy in the composite outcome group.

According to the final outcome, patients were classified into two groups - discharge (n = 231, 86.19%) or mortality (n = 37, 13.81%) (**Table 1**). The median age and duration of hospitalization for the all-cause mortality group were significantly higher compared to those of the discharge group (*p* = 0.002 and *p* = 0.031 respectively). Additionally, the prevalence of chronic pulmonary disease was markedly higher in the all-cause mortality group compared to the discharge group (*p* = 0.003). The incidence of critical COVID-19 cases was significantly higher in all-cause mortality group relative to the discharge group (all *p* < 0.05). However, no statistically significant difference was observed in the prevalence of severe COVID-19 between the two groups (*p* = 0.055). Despite not reaching a significance level of *p* < 0.05, there was an observable trend suggesting a potential association between severe COVID-19 and patient mortality. Furthermore, the rates of interventions such as oxygen/face mask oxygen (NC/FM), high-flow oxygen/noninvasive ventilation (HF/NIV), and tracheal intubation (TI) were significantly higher in the all-cause mortality group compared to the discharge group (all *p* < 0.001). In addition, the administration of antibiotics, anticoagulants, and immunoglobulin treatment was more frequent in the all-cause mortality group (all *p* < 0.05).

### 3.2 Laboratory findings and lymphocyte subsets of COVID-19 patients at hospital admission

**Figure 1:**
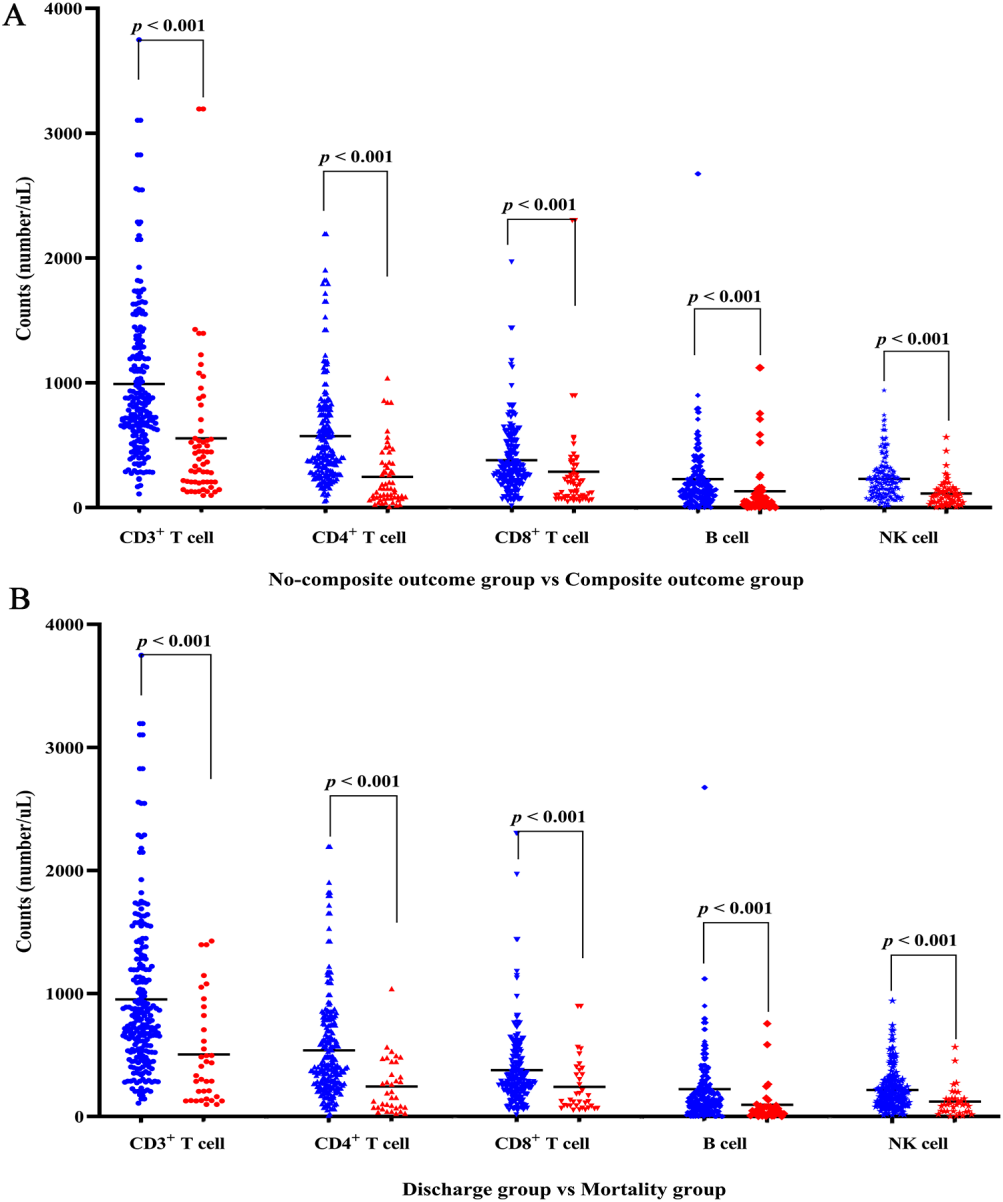
Lymphocyte subsets levels of COVID-19 patients with disease progression and final outcome. **(A)** Differences of lymphocyte subsets between non-composite outcome and composite outcome group. **(B)** Differences of lymphocyte subsets between discharge and mortality group. Red denotes composite outcome or mortality group; blue denotes non-composite outcome or discharge group.

The detailed characteristics of immune cells and lymphocyte subpopulations profile on admission of the COVID-19 patients were presented in **Table 2**. The composite outcome group and all-cause mortality group demonstrated a statistically significant reduction in lymphocyte (L), monocyte (M), and platelet (PLT) counts. Conversely, there was a notable increase in neutrophil count (N) and neutrophil-to-lymphocyte ratio (NLR) when compared to the non-composite outcome group and the discharge group, respectively (all *p* < 0.05). The quantification of lymphocyte subsets was conducted using flow cytometry measurement (**Table 2**). The counts of CD3^+^ T cell, CD4^+^ T cell, CD8^+^ T cell, B cell, and NK cell in both the composite outcome group (**Figure 1A**) and mortality group (**Figure 1B**) were significantly lower than the non-composite outcome group and the discharge group (all *p* < 0.001).

**Table 2:** Comparison of laboratory findings in COVID-19 patients stratified by disease progression and clinical outcome.

	All (n = 268)	Composite outcome (n = 66)	Non-composite outcome (n = 202)	*p*-Value	Death (n = 37)	Discharge (n = 231)	*p*-Value
Immune cells and lymphocyte subpopulations profile							
WBC (×10^9^/L)	6.61(4.84–9.03)	7.20(5.62–10.11)	6.60(4.53–8.90)	0.142	7.44(5.64–10.85)	6.56(4.68–8.92)	0.079
Neutrophil (×10^9^/L)	4.97(3.03–7.21)	6.41(3.95–8.22)	4.61(2.89–6.81)	0.023	6.30(3.98–8.36)	4.73(2.91–6.90)	0.020
Lymphocyte (×10^9^/L)	0.93(0.61–1.43)	0.59(0.30–0.89)	1.03(0.73–1.58)	< 0.001	0.59(0.35–0.93)	0.98(0.68–1.49)	< 0.001
NLR	4.87(2.65–9.46)	10.54(5.40–18.03)	4.04(2.39–7.28)	< 0.001	10.87(5.85–19.60)	4.29(2.59–8.50)	< 0.001
Monocyte (×10^9^/L)	0.43(0.29–0.60)	0.30(0.19–0.54)	0.44(0.31–0.64)	0.038	0.28(0.18–0.53)	0.44(0.30–0.63)	0.002
Platelet (×10^9^/L)	188.00(140.00–248.00)	163.50(119.50–216.00)	196.50(151.50–261.00)	< 0.001	142.00(117.00–183.00)	195.00(150.00–256.00)	< 0.001
CD3^+^ T cell (/uL)	724.50(452.00–1192.00)	377.50(205.00–546.00)	822.50(615.50–1285.00)	< 0.001	370.00(148.00–792.00)	775.00(525.00–1218.00)	< 0.001
CD4^+^ T cell (/uL)	387.00(232.00–650.00)	155.50(76.50–356.00)	434.00(282.50–748.50)	< 0.001	173.00(74.00–421.00)	404.00(264.00–742.00)	< 0.001
CD8^+^ T cell (/uL)	285.00 (183.00–469.00)	176.50(87.00–339.50)	302.50(214.50–526.00)	< 0.001	128.50(86.00–366.00)	291.00(203.00–489.00)	< 0.001
CD4^+^/CD8^+^ cell	1.41(0.89–2.24)	1.01(0.47–1.61)	1.50(0.97–2.34)	< 0.001	1.00(0.48–1.52)	1.49(0.94–2.34)	0.001
B cell (/uL)	131.00(58.00–242.00)	50.50(25.50–112.50)	157.00(87.00–265.50)	< 0.001	62.00(27.00–88.00)	145.00(86.00–261.00)	< 0.001
NK cell (/uL)	163.00(87.00–261.00)	91.50(42.50–141.50)	199.00(111.00–291.00)	< 0.001	97.00(42.00–146.00)	193.00(94.00–282.00)	< 0.001
Inflammatory markers							
CRP (mg/L)	35.27 (9.64–81.00)	61.10(34.30–120.60)	26.70(5.40–73.38)	< 0.001	60.93(40.32–119.94)	30.26(7.53–74.81)	< 0.001
SAA (mg/L)	119.00(23.30–249.76)	246.62(145.85–313.31)	68.52(14.31–211.30)	< 0.001	273.70(149.79–300.14)	84.44(17.03–213.86)	< 0.001
ESR (mm/H)	26.00(15.50–41.00)	36.00(21.00–47.00)	23.50(15.00–37.00)	0.007	27.50(17.00–46.00)	25.00(15.00–40.00)	0.756
PCT (ng/mL)	0.12(0.06–0.21)	0.14(0.09–0.84)	0.12(0.06–0.20)	0.227	0.14(0.09–0.97)	0.12(0.06–0.21)	0.011
Ferritin (ng/ml)	469.00(268.80–769.00)	663.00(276.00–883.50)	434.00(252.50–712.50)	0.094	693.00(482.00–1022.00)	439.00(247.00–746.00)	0.015
IL-6 (pg/ml)	9.50(4.80–36.50)	16.43(5.24–69.80)	9.34(4.22–30.61)	0.049	39.43(6.63–106.62)	9.28(4.22–30.30)	0.010
Coagulation indicators							
D-dimer (mg/L)	1.32(0.87–2.06)	1.69(1.12–3.20)	1.21(0.83–1.96)	0.001	1.82(1.18–3.23)	1.22(0.84–1.98)	0.003
Fibrinogen (mg/L)	4.70(3.64–5.96)	4.55(3.85–6.70)	4.70(3.63–5.96)	0.633	5.24(4.01–6.87)	4.66(3.63–5.96)	0.161
Cardiac function							
CK (U/L)	48.00(30.00–89.00)	41.00(25.50–121.00)	50.00(31.00–89.00)	0.686	58.00(38.00–236.00)	48.00(30.00–88.00)	0.138
CK-MB (U/L)	1.00(0.60–1.60)	1.50(0.63–2.15)	0.90(0.60–1.40)	0.001	1.60(0.84–3.05)	0.90(0.60–1.46)	< 0.001
cTn-I	7.60(2.60–19.30)	20.50(8.20–45.90)	6.10(2.20–15.12)	< 0.001	26.70(10.30–86.30)	6.35(2.20–16.48)	< 0.001
LDH (U/L)	252.00(204.00–342.00)	357.50(271.50–461.00)	235.00(197.00–292.00)	< 0.001	426.00(309.00–539.00)	245.00(198.00–307.00)	< 0.001
BNP	47.51(19.11–121.5)	35.87(16.11–89.42)	121.11(32.59–267.49)	< 0.001	186.93(93.00–344.27)	37.35(17.67–99.97)	< 0.001
Hepatorenal function							
Albumin (g/L)	36.06(32.36–43.32)	33.36(30.59–36.52)	37.28(32.96–46.50)	0.001	33.55(31.42–38.10)	36.59(32.60–43.96)	0.074
AST (U/L)	23.00(18.00–33.25)	30.00(22.00–44.00)	21.00(18.00–31.00)	0.814	38.00(26.00–49.00)	22.00(18.00–32.00)	< 0.001
ALT (U/L)	21.00(14.00–35.00)	23.00(13.00–37.50)	21.00(14.00–33.50)	0.004	24.00(14.00–38.00)	20.50(14.00–35.00)	0.312
Renal function							
BUN (mmol/L)	5.83(4.59–7.76)	7.16(5.27–11.59)	5.48(4.51–7.19)	< 0.001	9.50(5.83–14.31)	5.50(4.47–7.21)	< 0.001
Creatinine (μmol/L)	62.00(50.00–79.00)	65.50(51.50–111.00)	61.00(50.00–76.00)	0.016	84.00(57.00–125.00)	61.00(50.00–76.00)	< 0.001

WBC, white blood cell; NLR, neutrophil-to-lymphocyte ratio; CRP, C-reactive protein; SAA, serum amyloid A; ESR, erythrocyte sedimentation rate; PCT, procalcitonin; IL-6, interleukin-6; CK, creatine kinase; CK-MB, creatine kinase-MB; cTn-I, cardiac troponin-I; LDH, lactate dehydrogenase; BNP, brain natriuretic peptide; AST, aspartate aminotransferase; ALT, alanine aminotransferase; BUN, blood urea nitrogen.

Inflammatory markers, along with the indicators of coagulation, cardiac function, and hepatorenal function were assessed in the enrolled patients (**Table 2**). Notable statistical differences were observed between the non-composite outcome group and the composite outcome group for the following parameters: C-reactive protein (CRP), serum amyloid A (SAA), erythrocyte sedimentation rate (ESR), interleukin-6 (IL-6), D-dimer, creatine kinase-MB (CK-MB), cardiac troponin-I (cTn-I), lactate dehydrogenase (LDH), brain natriuretic peptide (BNP), albumin (ALB), alanine aminotransferase (ALT), blood urea nitrogen (BUN) and creatinine (Cr) (all *p* < 0.05). Additionally, a statistical difference was noted between the discharge and mortality groups for the following parameters: CRP, SAA, procalcitonin (PCT), ferritin, IL-6, D-dimer, CK-MB, cTn-I, LDH, BNP, aspartate aminotransferase (AST), ALB, BUN, and Cr (all *p* < 0.05).

### 3.3 Correlation between lymphocyte subsets and clinical characteristics in COVID-19 patients at hospital admission

**Figure 2:**
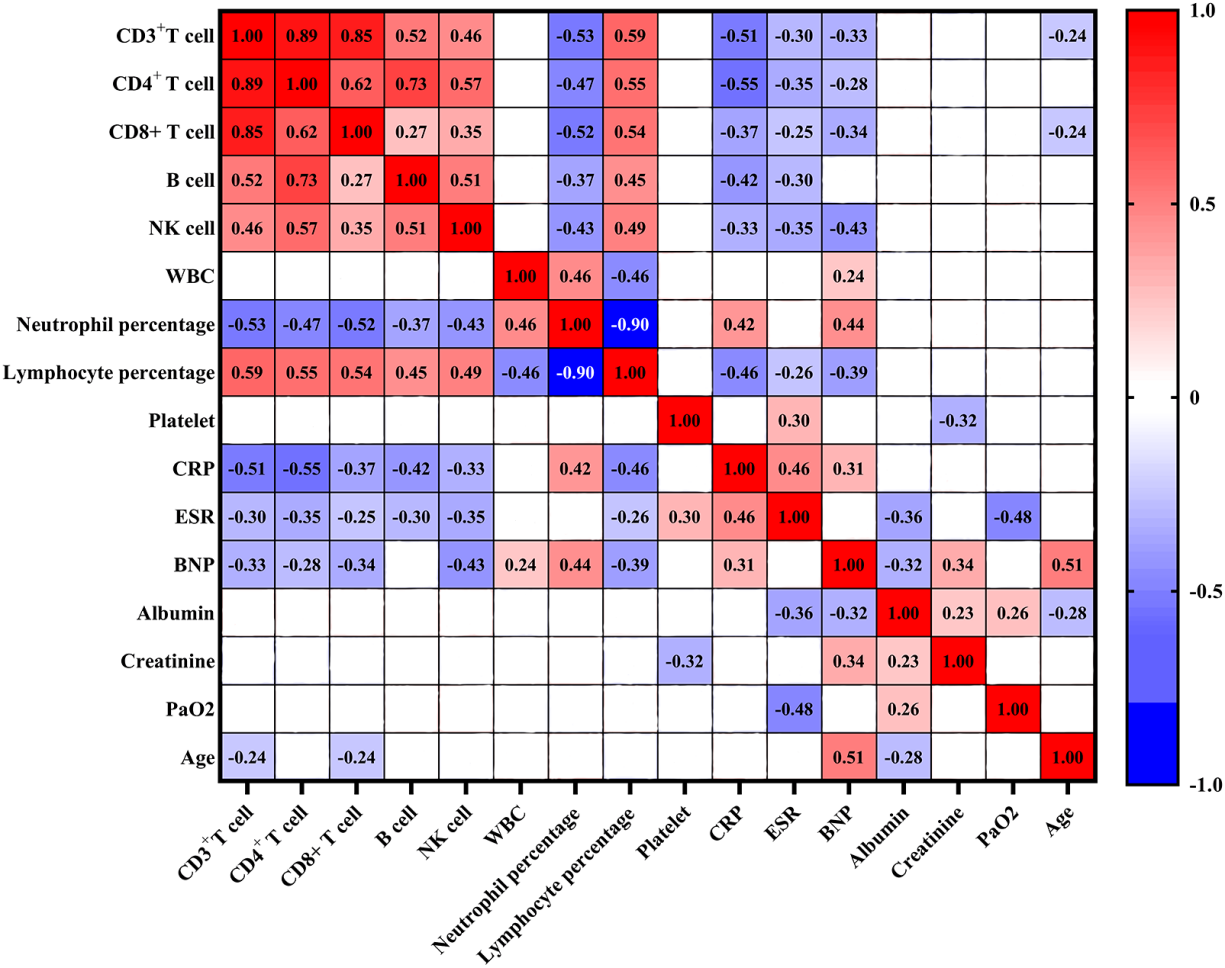
Correlation analysis between lymphocyte subsets and clinical characteristics in COVID-19 patients. Red denotes positive correlation, blue denotes negative correlation, and blank denotes no statistical significance. WBC: white blood cell; CRP, C-reactive protein; ESR, erythrocyte sedimentation rate; BNP, brain natriuretic peptide.

The counts of CD3^+^ T cell, CD4^+^ T cell, CD8^+^ T cell, B cell and NK cell exhibited positive correlations with lymphocyte percentage (all *p* < 0.05) (**Figure 2**). Conversely, these cell counts exhibited negative associations with neutrophil percentage, CRP, and ESR (all *p* < 0.05). Additionally, the levels of BNP were negatively correlated with the counts of CD3^+^ T cell, CD4^+^ T cell, CD8^+^ T cell, and NK cell (all *p* < 0.05), while no significant association was observed between B cell count and BNP levels. Furthermore, the counts of CD3^+^ T cell, CD4^+^ T cell, CD8^+^ T cell, B cell, and NK cell did not show significant associations with white blood cell (WBC) counts, platelet counts, albumin levels, creatinine levels, and PaO_2_.

### 3.4 ROC curve of lymphocyte subsets for the composite outcome and all-cause mortality in COVID-19 patients

**Figure 3:**
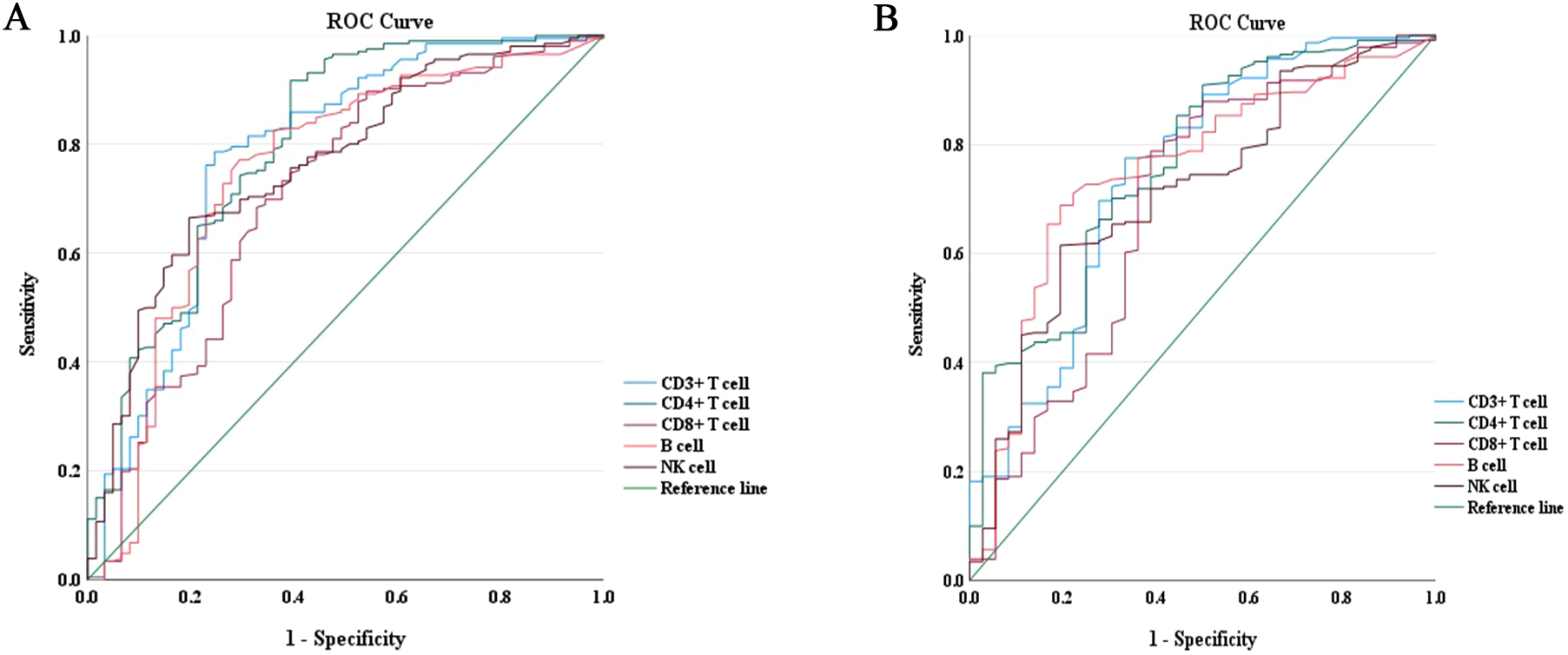
ROC curves of lymphocyte subsets for the prediction of disease progression and all-cause mortality in COVID-19 patients during hospitalization. **(A)** ROC curves of lymphocyte subsets for the prediction of disease progression. **(B)** ROC curves of lymphocyte subsets for the prediction of all-cause mortality. ROC, receiver operating characteristic.

The predictive power of lymphocyte subsets for predicting the composite outcome and all-cause mortality in enrolled COVID-19 patients was evaluated using receiver operating curve (ROC)/area under the curve (AUC) through plotting sensitivity against specificity. As depicted in **Figure 3A**, the AUC derived from CD4^+^ T cell count demonstrated superior predictive value for the composite outcome compared to other lymphocyte subsets cells. The ROC curve for CD4^+^ T cell count, as presented in **Figure 3A**, yielded an AUC of 0.801 (95% CI = 0.731–0.871). Employing the maximum Youden index, an optimal cut-off value of 203.50 cells/μl was determined with a sensitivity of 91.70% and specificity of 60.70% (**Table 3**). Similarly, as shown in **Figure 3B**, AUC derived from CD4^+^ T cell count exhibited the highest predictive value for all-cause mortality among different lymphocyte subsets. The ROC curve for CD4^+^ T cell count in assessing all-cause mortality is depicted in **Figure 3B**, yielding an AUC value of 0.765 (95% CI = 0.679–0.850). Utilizing the maximum Youden index, an optimal cut-off value of 156.00 cells/μl was identified with a sensitivity of 90.90% and specificity of 50.00% (**Table 3**).

**Table 3:** Comparison of lymphocyte subsets for predicting composite outcomes and mortality in hospitalized COVID-19 patients.

	AUC (95%CI)	Sensitivity	Specificity	Youden index	*p*-Value
Composite outcome					
CD3^+^ T cell (cells/μl)	0.781(0.707–0.856)	78.6%	75.4%	565.50	< 0.001
CD4^+^ T cell (cells/μl)	0.801(0.731–0.871)	91.3%	60.7%	203.50	< 0.001
CD8^+^ T cell (cells/μl)	0.705(0.624–0.785)	68.4%	67.2%	244.50	< 0.001
B cell (cells/μl)	0.749(0.670–0.827)	77.2%	70.5%	83.50	< 0.001
NK cell (cells/μl)	0.765(0.697–0.832)	66.5%	80.3%	147.50	< 0.001
All-cause mortality					
CD3^+^ T cell (cells/μl)	0.747(0.652–0.842)	77.5%	33.3%	504.00	< 0.001
CD4^+^ T cell (cells/μl)	0.765(0.679–0.850)	90.9%	50.0%	156.00	< 0.001
CD8^+^ T cell (cells/μl)	0.687(0.582–0.792)	78.8%	61.1%	188.50	< 0.001
B cell (cells/μl)	0.751(0.664–0.838)	68.8%	80.6%	90.50	< 0.001
NK cell (cells/μl)	0.713(0.624–0.802)	61.5%	80.6%	147.50	< 0.001

AUC, area under the curve.

### 3.5 Kaplan-Meier analysis of the association between CD4^+^ T cell count and all-cause mortality in COVID-19 patients

**Figure 4:**
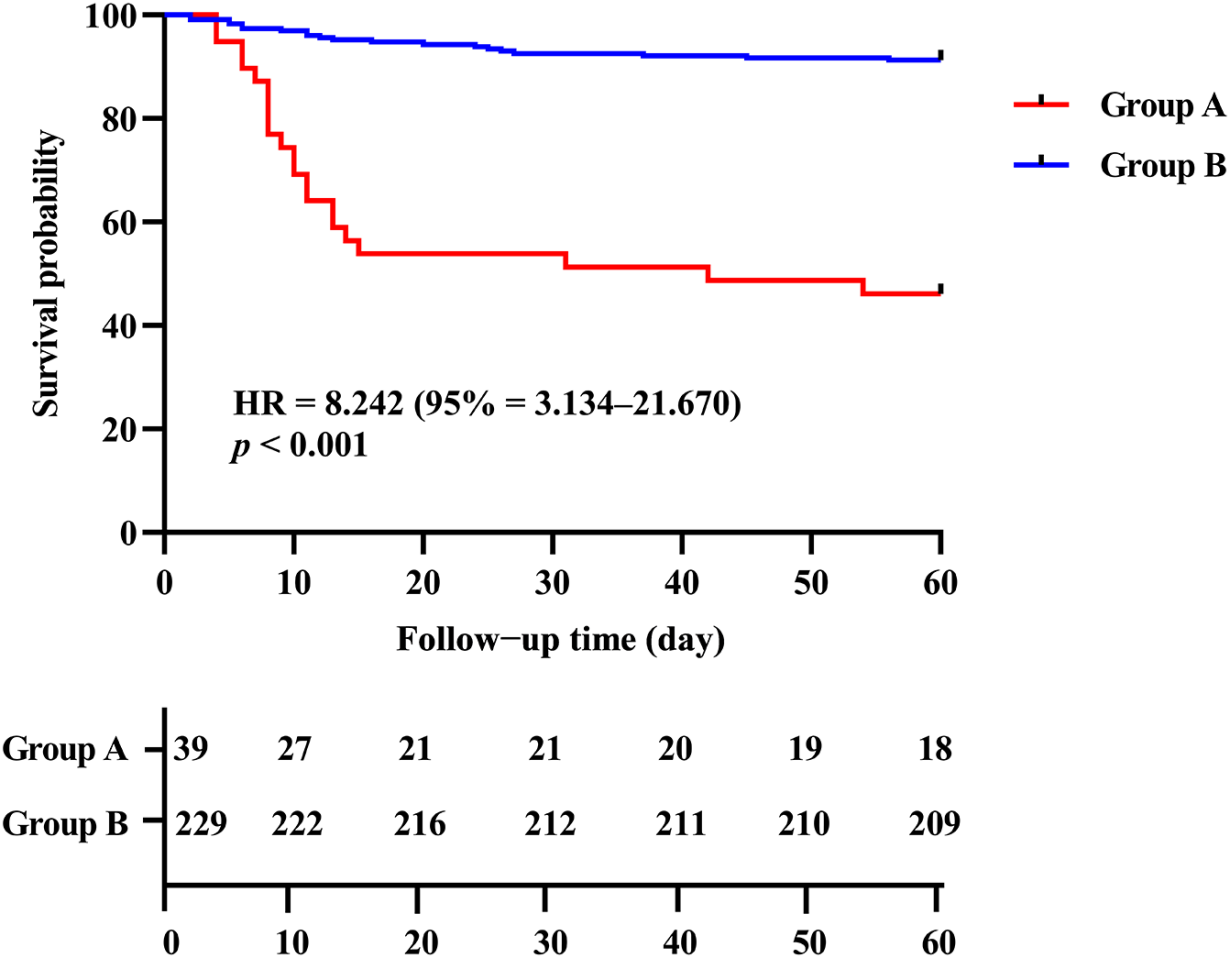
Kaplan-Meier analysis of the association between CD4^+^ T cell count level and the all-cause mortality in COVID-19 patients. Group A: CD4^+^ T cell count < 156.00 cells/μl; Group B: CD4^+^ T cell count ≥ 156.00 cells/μl.

Based on the ROC curve analysis for all-cause death, the optimal cutoff value for CD4^+^ T cell count was determined as 156.00 cells/μl using Youden’s index. Subsequently, the enrolled patients were categorized into two distinct groups: Group A comprised individuals with CD4^+^ T cell count below 156.00 cells/μl, while Group B consisted of those with CD4^+^ T cell count equal to or exceeding 156.00 cells/μl. The enrolled patients were followed for a period of 60 days from the time of admission. The Kaplan-Meier curves revealed a significantly higher probability of mortality in patients with low CD4^+^ T cell count (< 156.00 cells/μl) compared to those with high CD4^+^ T cell count (≥ 156.00 cells/μl) (log-rank *p* < 0.001, HR = 8.242, 95% CI = 3.134–21.670) (**Figure 4**).

### 3.6 Multivariate logistic regression analysis of the risk factors for disease progression and all-cause mortality in COVID‐19 patients

A logistic regression analysis was conducted to identify lymphocyte subsets associated with composite outcome and mortality in COVID‐19 patients treated with azvudine (**Table 4**). The univariable analyses revealed that low levels of CD3^+^ T cell, CD4^+^ T cell, CD8^+^ T cell, B cell, and NK cell were significantly associated with disease progression and mortality in the enrolled patients (all *p* < 0.001) (**Table 4**). After adjusting for age, gender, comorbidities and severity at admission, the multivariate logistic regression analysis demonstrated that low levels of CD3^+^ cell (< 565.50 cells/μl), CD4^+^ cell (< 203.50 cells/μl), and NK cell (< 147.50 cells/μl) were significantly associated with disease progression in COVID-19 patients treated with azvudine (OR = 4.198, 95% CI = 1.155–15.262, *p* = 0.029; OR = 4.313, 95% CI = 1.645–11.307, *p* = 0.003; OR = 3.345, 95% CI = 1.436–7.791, *p* = 0.005; respectively) (**Table 4**). Additionally, the multivariate logistic regression analysis revealed that low CD4^+^ T cell count (< 156.00 cells/μl) was associated with all-cause mortality in COVID-19 patients treated with azvudine (OR = 5.860, 95% CI = 1.727–19.878, *p* = 0.005).

**Table 4:** Univariate and multivariate analyses of lymphocyte subsets for predicting the composite outcome and mortality in COVID-19 patients during hospitalization.

Variables	Univariate		Multivariate	
	OR (95% CI)	*p*-Value	OR (95% CI)	*p*-Value
Composite outcome				
CD3^+^ T cell count < 565.50 (cells/μl)	11.040(5.650–21.571)	< 0.001	4.198(1.155–15.262)	0.029
CD4^+^ T cell count < 203.50 (cells/μl)	17.230(8.346–35.194)	< 0.001	4.313(1.645–11.307)	0.003
CD8^+^ T cell count < 244.50 (cells/μl)	4.380(2.381–8.054)	< 0.001	0.588(0.184–1.886)	0.372
B cell count < 83.50 (cells/μl)	8.132(4.292–15.410)	< 0.001	1.790(0.759–4.220)	0.183
NK cell count < 147.50 (cells/μl)	8.167(4.079–16.352)	< 0.001	3.345(1.436–7.791)	0.005
All-cause mortality				
CD3^+^ T cell count < 504.00 (cells/μl)	6.755(3.166–14.410)	< 0.001	0.590(0.131–2.661)	0.493
CD4^+^ T cell count < 156.00 (cells/μl)	14.861(6.581–33.560)	< 0.001	5.860(1.727–19.878)	0.005
CD8^+^ T cell count < 188.50 (cells/μl)	5.440(2.601–11.375)	< 0.001	2.283(0.701–7.429)	0.170
B cell count < 90.50 (cells/μl)	7.778(3.380–17.900)	< 0.001	2.499(0.874–7.145)	0.087
NK cell count < 147.50 (cells/μl)	6.657(2.798–15.837)	< 0.001	2.359(0.821–6.775)	0.111

## 4 Discussion

This retrospective study aimed to provide a comprehensive analysis by investigating hospitalized patients treated with azvudine during the Omicron surge. We analyzed the demographic, clinical characteristics, laboratory findings, and lymphocyte subpopulations profile upon admission for a cohort 268 hospitalized COVID-19 patients treated with azvudine. Our study has shown that the counts of lymphocyte subsets were significantly reduced in patients with disease progression and mortality. Correlation analyses revealed negative associations between lymphocyte subset counts and levels of inflammatory markers. Furthermore, Kaplan-Meier curve and multivariate regression analysis demonstrated a significant association between low CD4^+^ T cell count and adverse outcome. The findings highlight the potential of CD4^+^ T cell as a novel predictive tool for COVID-19 patients treated with azvudine.

Multiple studies have now established that the dysregulated immune responses and hyperinflammation induced by SARS-CoV-2 can result in detrimental tissue damage, both locally and systemically (21, 22). Notably, several symbol inflammatory cytokines, including CRP, SAA, ESR, PCT, ferritin, and IL-6, were significantly higher in the composite outcome and mortality group compared to the non-composite outcome and discharge group. Correlation analysis showed that lymphocyte subset counts exhibited negative associations with the levels of these inflammatory markers. Meanwhile, a previous study has also reported a negative correlation between lymphocyte subset counts and levels of inflammatory markers in the context of the Omicron variant, which was consistent with our findings (23). These findings suggest that dysregulated immune responses may play a pivotal role in the pathogenesis of COVID-19 patients, particularly concerning disease progression and mortality.

A substantial proportion of individuals contracted SARS-CoV-2 infection during the Omicron surge in the following months. However, there exists an insufficiency in the availability of antiviral medications to adequately meet the demand from COVID-19 patients during this period. On July 25, 2022, the National Medical Products Administration has conditionally authorized the utilization of azvudine for the treatment of COVID-19. Based on current clinical evidence, azvudine demonstrates potential in reducing in-hospital mortality among COVID-19 patients, particularly within the severe and critical cases (17–19). Nevertheless, there is a lack of reports on the lymphocyte subpopulations profile in relation to disease progression as well as mortality among COVID-19 patients receiving azvudine treatment. A previous study demonstrated a significant association between low levels of CD8^+^ T cell (< 201 cells/μl) and an increased risk of composite outcome; additionally, low levels of CD4^+^ T cell (< 368 cells/μl) and CD8^+^ T cell (< 201 cells/μl) were closely associated with the mortality outcome in COVID‐19 patients receiving Nirmatrelvir therapy (24). Our study revealed that low levels of CD3^+^ cell (< 565.50 cells/μl), CD4^+^ cell (< 203.50 cells/μl), and NK cell (< 147.50 cells/μl) were associated with disease progression in COVID-19 patients receiving azvudine treatment. Furthermore, multivariate logistic regression analysis also demonstrated a robust association between low CD4^+^ T cell count (< 156.00 cells/μl) and mortality in COVID-19 patients undergoing azvudine treatment. Additionally, the present study demonstrated a significantly higher probability of mortality in patients with low CD4^+^ T cell count (< 156.00 cells/μl) compared to those with high CD4^+^ T cell count (≥ 156.00 cells/μl), as indicated by the Kaplan-Meier curves (HR = 8.242). Consequently, these findings offer valuable insights for physicians to optimize the use of azvudine.

Respiratory viral infections have been shown to impact the quantity and distribution of peripheral lymphocytes, with a significant proportion of patients experiencing lymphocytopenia during the acute phase of severe acute respiratory syndrome (SARS) (25). In the context of COVID-19, there has been an observed an upregulation in the expression levels of programmed cell death receptor 1 (PD-1) and Tim-3 within T lymphocytes, indicating a potential depletion of T cells (26). The immune response is closely related to the pathogenesis, progression, and prognosis of COVID-19 patients, particularly pertaining to the activation of adaptive immune function (23). CD4^+^ and CD8^+^ T cells represent the fundamental constituents of adaptive immunity, exhibiting diverse helper and effector functionalities, as well as the capacity to eliminate infected cells (27). A previous study involving 701 COVID-19 patients revealed a significant association between mortality and reduced counts of CD4^+^ T cells (≤ 500 cells/μl) (12). Additionally, Xu et al. reported a significant decrease in the counts of total lymphocytes, CD3^+^ T cells, CD4^+^ T cells, CD8^+^ T cells, B cells, and NK cells among 187 hospitalized patients with COVID-19. Sensitivity analysis further indicated that a low count of CD4^+^ T cells (< 100 cells/μl) was identified as a risk factor for mortality in COVID-19 patients (28). Multiple studies have documented a substantial decline in CD4^+^ T cell count as the severity of COVID-19 progresses (29, 30). Although the complete understanding of disease pathogenesis remains elusive, it is widely believed that an aberrant and hyperactive immune response plays a pivotal role in the development of severe COVID-19, potentially involving CD4^+^ T cells.

A recent investigation indicated that the CD4^+^ T cell response to SARS-CoV-2 is influenced by vaccination (31); however, China took longer to prevent and control SARS-CoV-2 compared to other countries, maintaining preventive measures until December 2022 when the government announced their cessation. At this moment, the majority of adults aged 18 and older had completed their COVID-19 vaccination regimen. Furthermore, most participants in this study had also received complete COVID-19 vaccination. Consequently, we did not provide detailed statistics regarding the vaccination status of the enrolled patients. Due to limitations in sample size, we did not conduct a cohort analysis based on their COVID-19 vaccination status of the enrolled patients. In addition, the study also lacked detailed statistical data on patients receiving immunosuppressive therapy for inflammatory autoimmune diseases or those receiving anti-rejection medications post-organ transplantation. A recent study revealed that a significant proportion of these patients did not develop detectable anti-SARS-CoV-2 IgG three months following the completion of their vaccination regimen (32). Therefore, future studies should focus on immunophenotyping characteristics and clinical outcomes of immunodeficient patients who received azvudine during the Omicron variant surge.

There are some limitations in our meta-analysis. Firstly, the retrospective design of the study presents a significant constraint, as it hinders establishing a causal relationship between lymphocyte subsets and poor outcomes in COVID-19 patients treated with azvudine. Requisite longitudinal studies are imperative to establish conclusive causal relationships in future research. Secondly, it should be noted that this study was conducted at a single center in Shandong province, which may limit the generalizability of the findings to the broader context of China. Further studies encompassing various geographical regions and ethnic populations are warranted to investigate the correlation between lymphocyte subsets and the prognosis of patients with COVID-19. Thirdly, data, including lymphocyte subsets counts, were obtained at the time of admission. However, we did not monitor the dynamic changes in lymphocyte subsets throughout the course of the disease. Lastly, there is no record and statistical analysis regarding the vaccination status of patients.

## 5 Conclusion

In conclusion, the current research demonstrated a significant correlation between decreased lymphocyte subset cell counts and disease progression as well as mortality in COVID-19 patients underwent azvudine treatment. A significant correlation was identified between low CD4^+^ T cell count level and adverse outcomes. Therefore, these findings may serve as valuable references for physicians to optimize the utilization of azvudine in clinical practice.

## Data Availability

The original contributions presented in the study are included in the article/supplementary material. Further inquiries can be directed to the corresponding authors.
